# Lymphoepithelial Cyst Presenting as a Lateral Neck Mass: A Diagnostic Pitfall in Adults

**DOI:** 10.7759/cureus.105294

**Published:** 2026-03-16

**Authors:** Oumayma Leassal, Cedric Ango, Hicham Attifi, Mounir Hmidi

**Affiliations:** 1 Otolaryngology - Head and Neck Surgery, Military Hospital Moulay Ismail, Meknes, MAR

**Keywords:** cystic neck mass, differential diagnosis, histopathology, lateral cervical mass, lymphoepithelial cyst, surgical excision

## Abstract

Cystic lateral neck masses in adults are most commonly attributed to branchial cleft cysts. Lateral cervical lymphoepithelial cyst is a rare benign entity that may represent a diagnostic challenge because of its nonspecific clinical presentation. We report the case of a 32-year-old woman presenting with a slowly enlarging right lateral cervical swelling evolving for one year. Clinical examination revealed a painless, well-defined, and mobile mass. Cervical ultrasonography followed by CT demonstrated a well-circumscribed cystic lesion measuring 37 × 45 × 65 mm, initially suggestive of a branchial cleft cyst. Complete surgical excision was performed, and histopathological examination confirmed the diagnosis of a lymphoepithelial cyst. HIV serology was negative. The postoperative course was uneventful with no recurrence. This case highlights the diagnostic challenges of cystic neck masses in adults and emphasizes the importance of histopathological confirmation and surgical excision for definitive diagnosis and management.

## Introduction

Cystic lateral neck masses represent a frequent diagnostic challenge in otorhinolaryngology. In adults, their etiology is most commonly related to branchial cleft cysts; however, other less frequent entities must also be considered, including lymphoepithelial cysts and cystic metastatic lymph nodes [1].

Lymphoepithelial cyst is a benign lesion characterized histologically by a cystic cavity lined with squamous epithelium surrounded by dense lymphoid tissue. Although most commonly described in the oral cavity and salivary glands, its occurrence in the lateral cervical region remains uncommon [2,3].

In adult patients, cystic neck masses require careful evaluation because they may mimic malignant lesions, particularly cystic metastases from human papillomavirus-related oropharyngeal carcinoma [1,4]. For this reason, the diagnostic approach to a lateral neck mass in adults should always be systematic and primarily aimed at excluding malignancy. This evaluation typically includes careful clinical examination, imaging studies such as ultrasonography and CT, and, when appropriate, fine-needle aspiration cytology to rule out metastatic disease before considering a benign congenital lesion. Imaging studies such as ultrasonography and CT help characterize the cystic nature of the lesion but are often insufficient for establishing a definitive diagnosis [5].

Definitive diagnosis relies on histopathological examination after surgical excision, which also represents the treatment of choice with excellent prognosis [2]. We report a case of a lateral cervical lymphoepithelial cyst in an immunocompetent adult patient to highlight the diagnostic difficulties and the importance of histological confirmation.

## Case presentation

**Figure 1:**
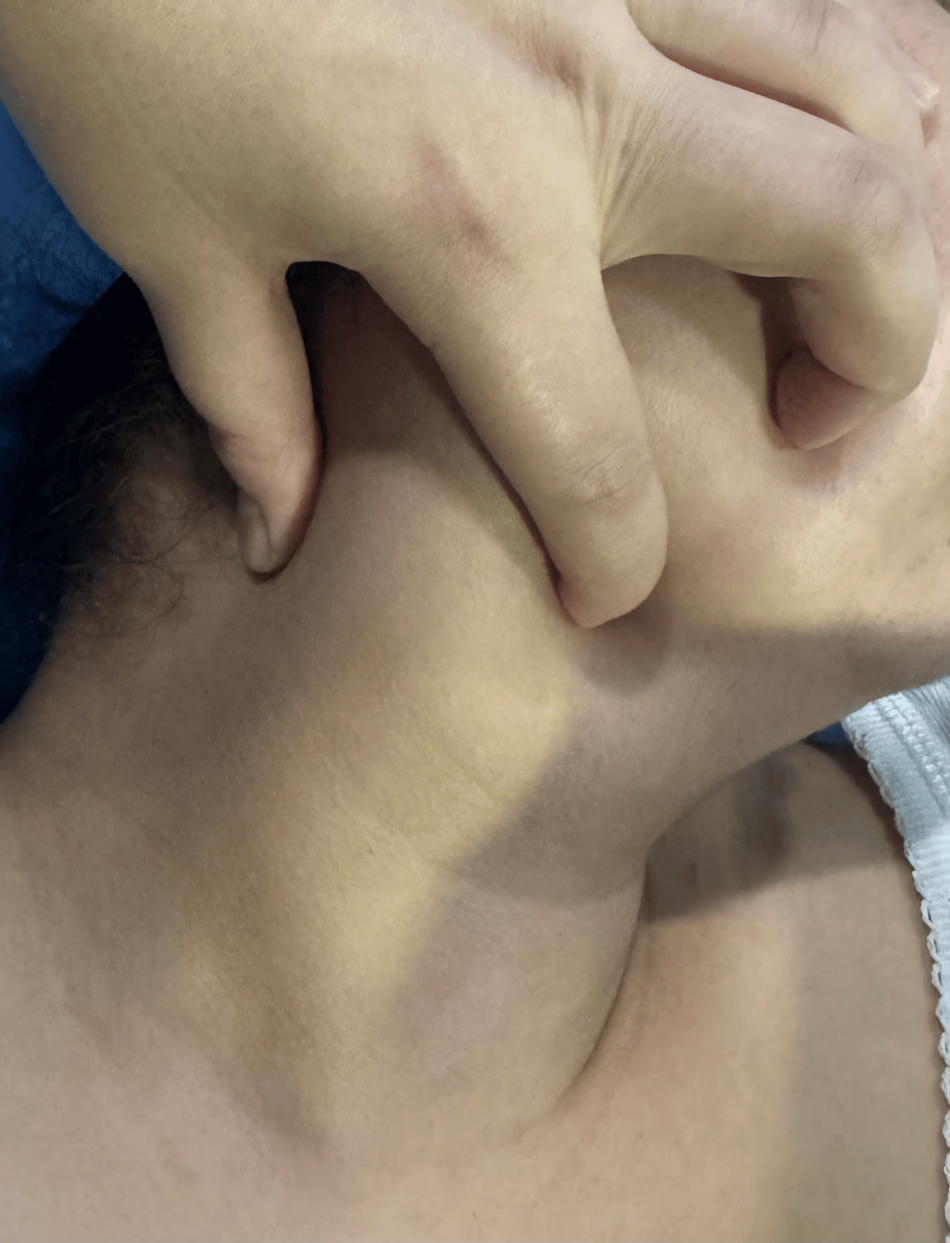
Preoperative clinical photograph showing a right lateral cervical mass.

A 32-year-old woman with no significant medical or surgical history was admitted to our department for evaluation of a right lateral cervical swelling evolving for approximately one year (Figure 1). The clinical history was characterized by a gradual and painless increase in the size of the mass, without associated symptoms such as dysphagia, dyspnea, dysphonia, cervical pain, fever, or general health deterioration.

Clinical examination revealed a right lateral neck mass located in the middle third of the neck, with regular contours and well-defined borders, measuring approximately 6 cm in its largest dimension. The mass was soft in consistency, non-pulsatile, and mobile relative to both superficial and deep planes, with no overlying skin inflammatory changes.

Complete otorhinolaryngological examination, including evaluation of the oral cavity, oropharynx, hypopharynx, and nasopharynx, revealed no associated abnormalities. Flexible endoscopic evaluation of the upper aerodigestive tract was also performed and did not reveal any suspicious lesions. No cervical lymphadenopathy was detected. Fine-needle aspiration cytology was not performed because imaging findings strongly suggested a benign cystic lesion, and complete surgical excision was planned for both diagnostic and therapeutic purposes.

**Figure 2:**
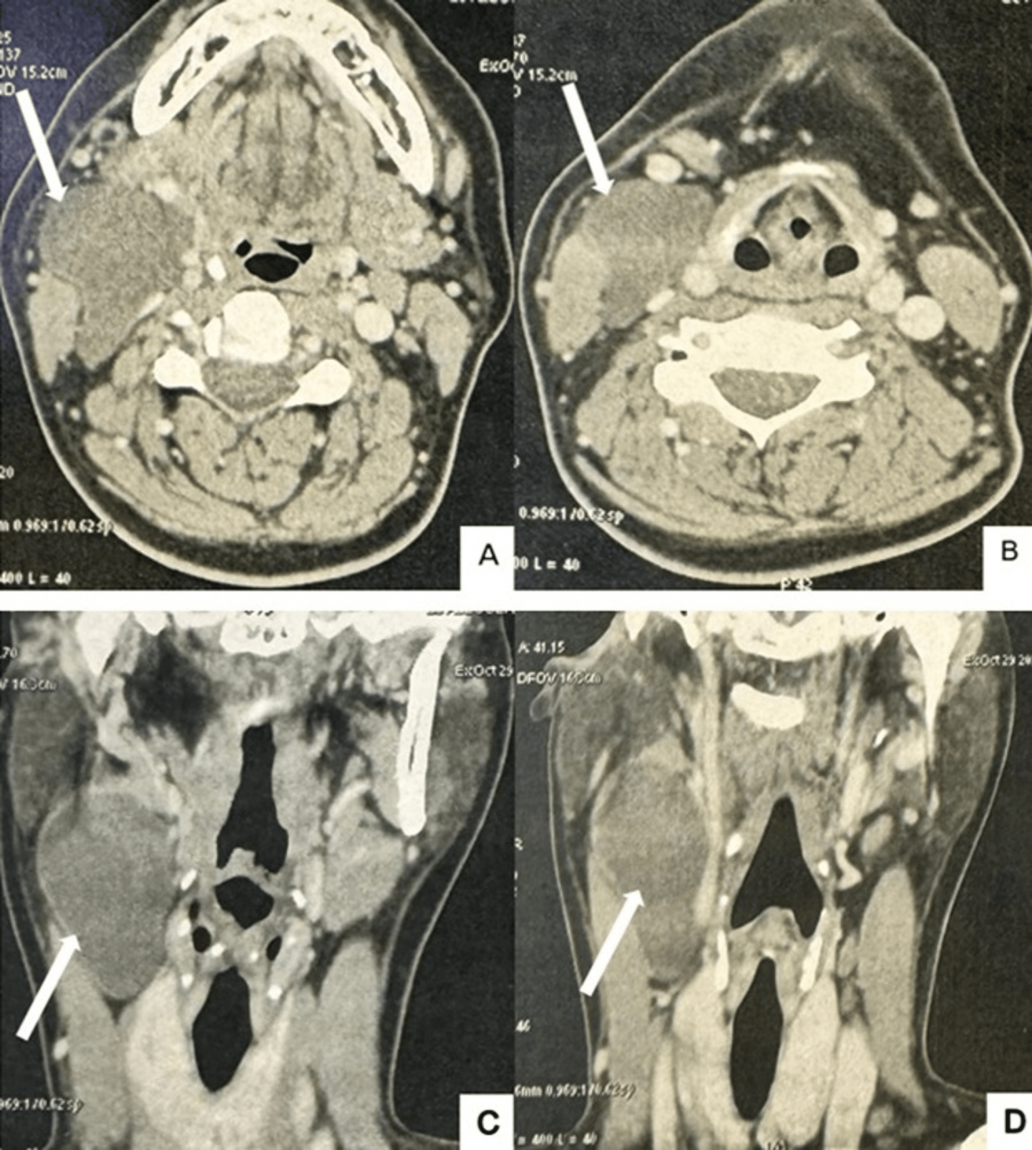
Contrast-enhanced cervical CT scan demonstrating a well-defined cystic lesion in the right lateral cervical region displacing adjacent structures without signs of invasion (arrows). (A, B) Axial views. (C, D) Coronal views.

Cervical ultrasonography demonstrated a well-circumscribed cystic lesion with a thin wall and homogeneous content in the right lateral cervical region. Contrast-enhanced cervical CT confirmed the presence of a hypodense cystic mass measuring 37 × 45 × 65 mm, without contrast enhancement, displacing adjacent structures without evidence of infiltration (Figure 2). Based on these clinical and radiological findings, a branchial cleft cyst was initially suspected.

**Figure 3:**
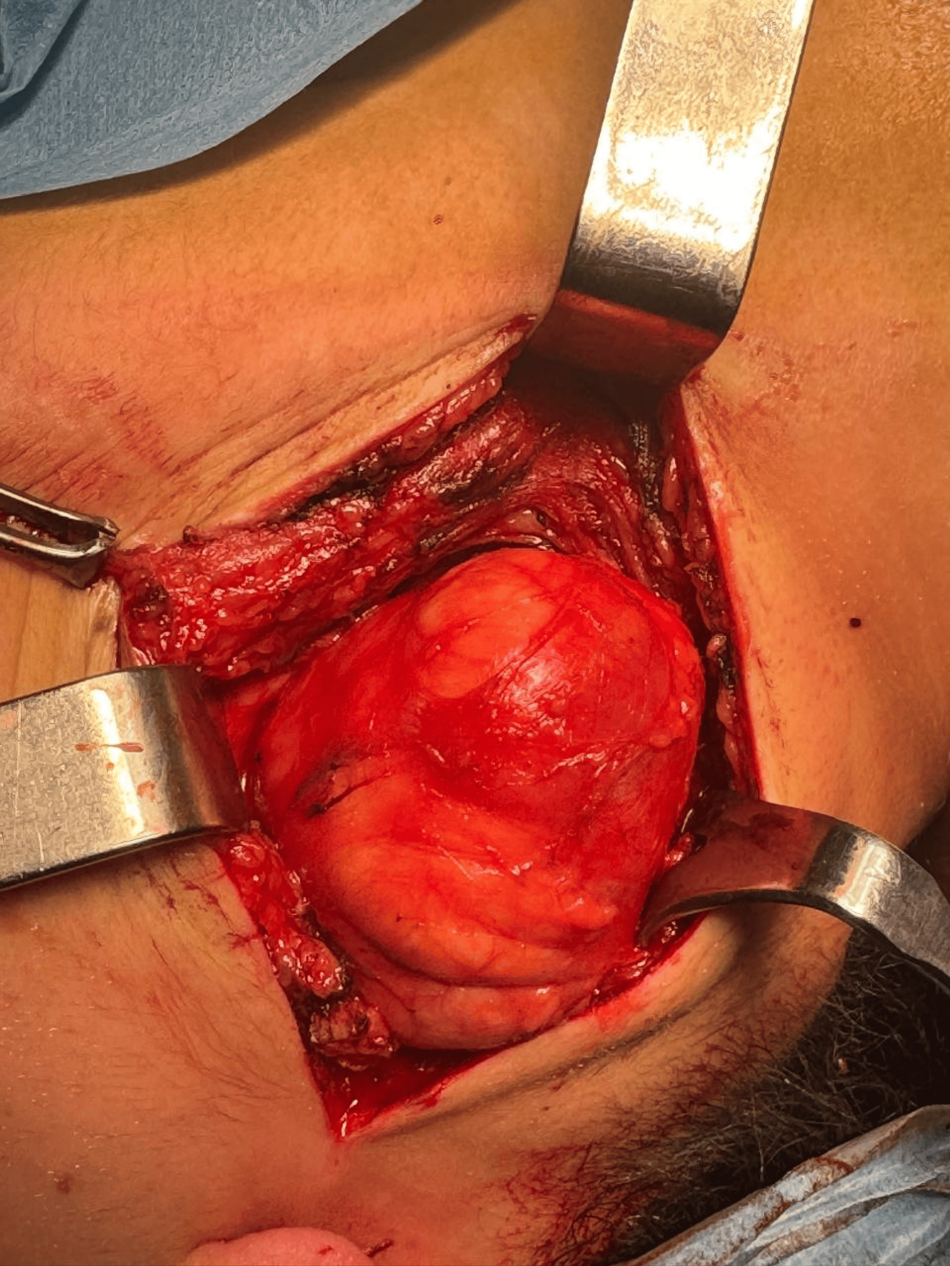
Intraoperative view showing exposure of the cystic mass in the right lateral cervical region.

**Figure 4:**
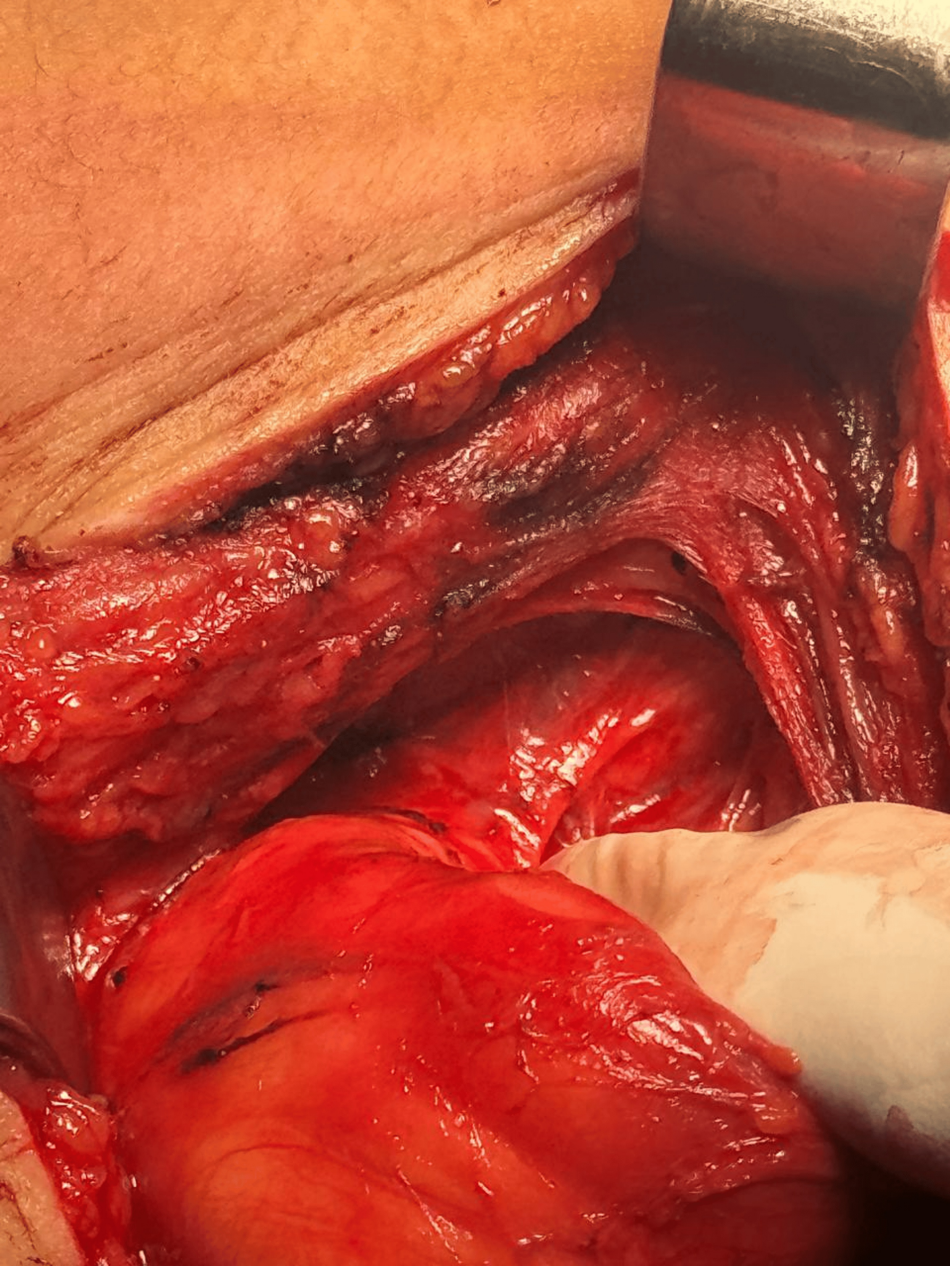
Intraoperative view showing progressive mobilization of the cystic mass before removal.

**Figure 5:**
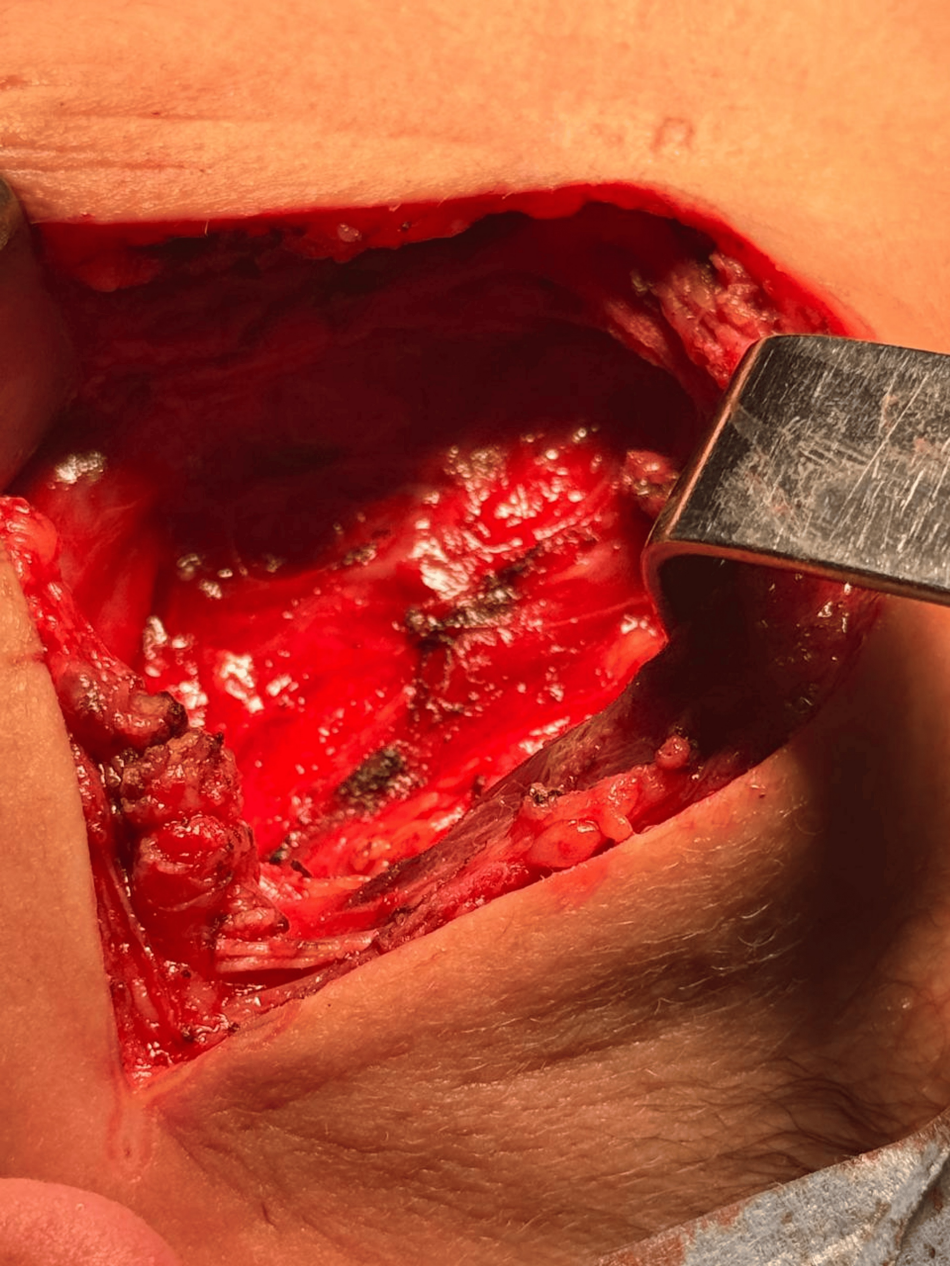
Surgical cavity after complete excision of the cystic lesion.

**Figure 6:**
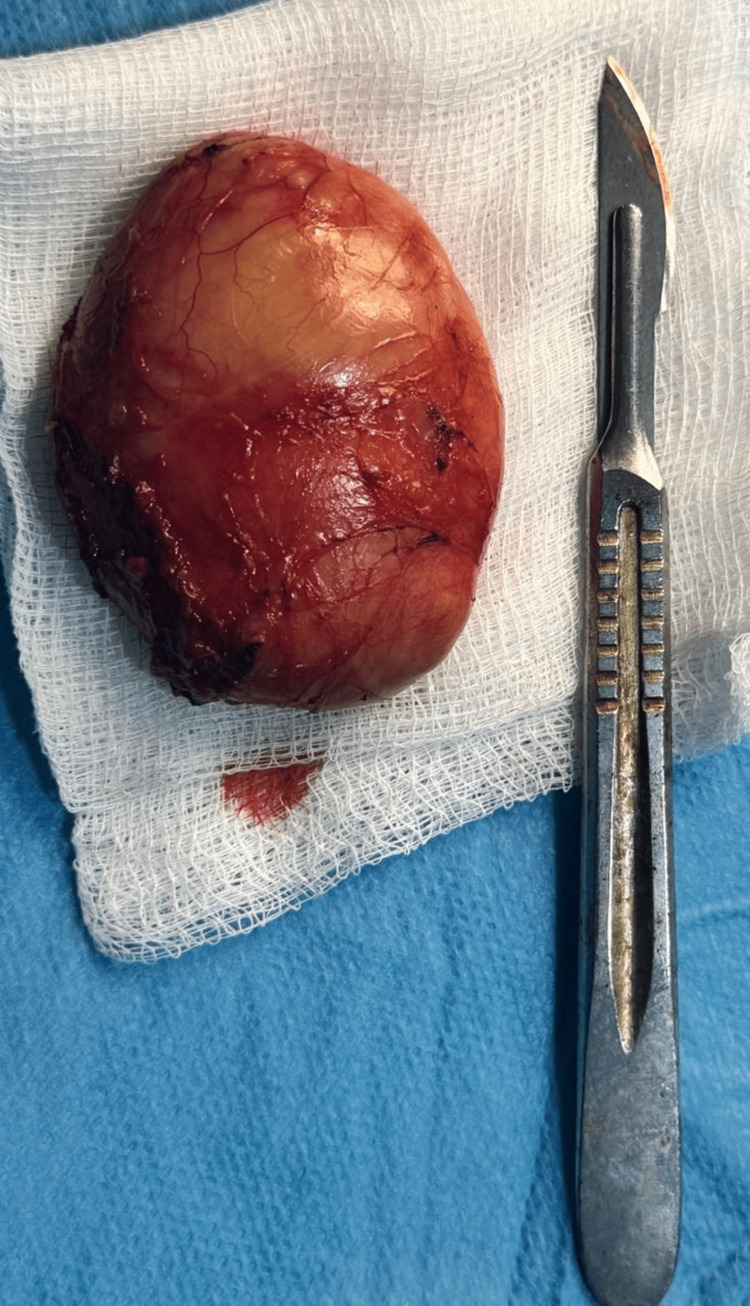
Excised cystic mass after complete surgical removal.

Surgical management was undertaken. The patient underwent a right lateral cervical incision with complete excision of the mass. Intraoperative findings revealed a well-encapsulated lesion with a thin wall containing clear fluid, easily dissected from adjacent neurovascular structures (Figures 3-6). No intraoperative complications were encountered, and the immediate postoperative course was uneventful.

**Figure 7:**
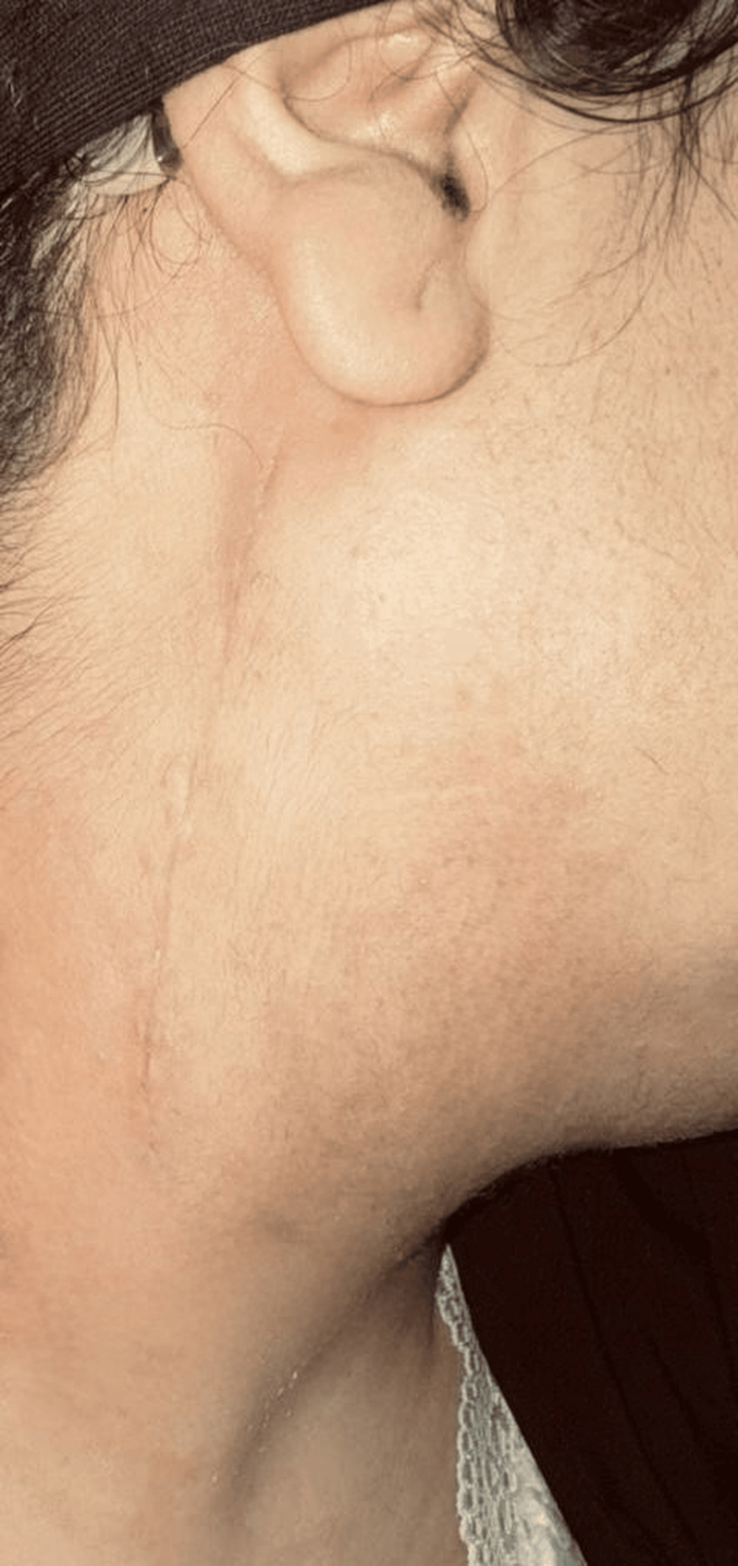
Postoperative cervical scar 15 days after surgery showing satisfactory healing.

Histopathological examination of the surgical specimen confirmed the diagnosis of a lymphoepithelial cyst, characterized by a cystic cavity lined by stratified squamous epithelium surrounded by dense lymphoid stroma containing germinal centers. In this context, HIV serology was performed and returned negative. At six months of follow-up, the patient remained asymptomatic with no evidence of recurrence (Figure 7).

## Discussion

Lymphoepithelial cysts are uncommon benign lesions that typically arise from epithelial inclusions within lymphoid tissue. They are most frequently described in the oral cavity, particularly in the floor of the mouth and the lateral border of the tongue, but may rarely occur in the cervical region, where they can present as a lateral neck mass and pose a diagnostic challenge [2,3].

In adults, the evaluation of a lateral neck mass requires careful consideration because cystic lesions may represent a wide spectrum of pathologies ranging from benign congenital anomalies to malignant neoplasms. Clinical guidelines emphasize the importance of a systematic approach combining clinical examination, imaging, and histopathological confirmation to establish an accurate diagnosis [1].

Cervical lymphoepithelial cysts are often clinically indistinguishable from other cystic neck lesions, particularly branchial cleft cysts, which represent the most common congenital cystic masses of the lateral neck. This overlap frequently leads to diagnostic confusion, especially in adult patients, where cystic metastatic lymph nodes must also be considered in the differential diagnosis [6].

It is important to note that the distinction between lymphoepithelial cysts and branchial cleft cysts remains debated in the literature. While some authors consider lymphoepithelial cysts to represent a variant of branchial cleft cysts, others describe them as distinct entities based on differences in embryological origin and histopathological features.

Imaging studies play a crucial role in the preoperative evaluation of cystic cervical lesions. Ultrasound is usually the first-line examination, while CT and MRI provide better characterization of lesion boundaries and their relationship with adjacent anatomical structures. These imaging modalities typically demonstrate a well-defined cystic lesion with homogeneous fluid content, although imaging alone is often insufficient to establish a definitive diagnosis [5,7-10].

Histopathological examination remains the gold standard for diagnosis. Lymphoepithelial cysts are characterized by a cystic cavity lined by stratified squamous epithelium surrounded by dense lymphoid tissue that may contain germinal centers. This histological pattern is essential to distinguish them from other cystic lesions of the neck [2,6].

In some clinical contexts, lymphoepithelial cysts have also been associated with viral infections, particularly in patients infected with HIV, where they may involve the salivary glands and cervical lymphoid tissue. However, similar lesions can also occur in immunocompetent individuals without any underlying systemic disease [4,11].

The treatment of choice for cervical lymphoepithelial cysts is complete surgical excision. Surgery allows both definitive diagnosis and curative treatment, with a very low risk of recurrence when the lesion is entirely removed. In most reported cases, postoperative outcomes are favorable, and complications are rare [7,12].

Our case illustrates the diagnostic difficulty posed by cystic lateral neck masses in adults. The clinical and radiological features initially suggested a congenital cystic lesion, whereas the final diagnosis relied exclusively on histopathological examination. This highlights the importance of considering lymphoepithelial cysts in the differential diagnosis of lateral cervical masses, even though they remain a relatively rare entity [3,13].

## Conclusions

Lymphoepithelial cyst is a rare benign lesion that can present as a lateral neck cystic mass and may mimic other cervical cystic lesions, particularly branchial cleft cysts. This case highlights the diagnostic challenge posed by such lesions in adults and emphasizes the importance of considering lymphoepithelial cyst in the differential diagnosis of lateral cervical cystic masses. Although imaging studies can suggest a benign cystic lesion, histopathological examination remains essential for establishing the definitive diagnosis. As this report describes a single case, further studies are needed to better characterize the clinical presentation and diagnostic features of lymphoepithelial cysts in adults.
